# Molecular epidemiology of *Staphylococcus aureus* bacteremia in a single large Minnesota medical center in 2015 as assessed using MLST, core genome MLST and *spa* typing

**DOI:** 10.1371/journal.pone.0179003

**Published:** 2017-06-02

**Authors:** Kyung-Hwa Park, Kerryl E. Greenwood-Quaintance, James R. Uhl, Scott A. Cunningham, Nicholas Chia, Patricio R. Jeraldo, Priya Sampathkumar, Heidi Nelson, Robin Patel

**Affiliations:** 1 Division of Clinical Microbiology, Department of Laboratory Medicine and Pathology, Mayo Clinic, Rochester, Minnesota, United States of America; 2 Department of Infectious Diseases, Chonnam National University Medical School, Gwangju, South Korea; 3 Center for Individualized Medicine, Mayo Clinic, Rochester, Minnesota, United States of America; 4 Department of Surgery, Mayo Clinic, Rochester, Minnesota, United States of America; 5 Division of Infectious Diseases, Department of Internal Medicine, Mayo Clinic, Rochester, Minnesota, United States of America; Rockefeller University, UNITED STATES

## Abstract

*Staphylococcus aureus* is a leading cause of bacteremia in hospitalized patients. Whether or not *S*. *aureus* bacteremia (SAB) is associated with clonality, implicating potential nosocomial transmission, has not, however, been investigated. Herein, we examined the epidemiology of SAB using whole genome sequencing (WGS). 152 SAB isolates collected over the course of 2015 at a single large Minnesota medical center were studied. *Staphylococcu*s protein A (*spa*) typing was performed by PCR/Sanger sequencing; multilocus sequence typing (MLST) and core genome MLST (cgMLST) were determined by WGS. Forty-eight isolates (32%) were methicillin–resistant *S*. *aureus* (MRSA). The isolates encompassed 66 *spa* types, clustered into 11 *spa* clonal complexes (CCs) and 10 singleton types. 88% of 48 MRSA isolates belonged to *spa* CC-002 or -008. Methicillin-susceptible *S*. *aureus* (MSSA) isolates were more genotypically diverse, with 61% distributed across four *spa* CCs (CC-002, CC-012, CC-008 and CC-084). By MLST, there was 31 sequence types (STs), including 18 divided into 6 CCs and 13 singleton STs. Amongst MSSA isolates, the common MLST clones were CC5 (23%), CC30 (19%), CC8 (15%) and CC15 (11%). Common MRSA clones were CC5 (67%) and CC8 (25%); there were no MRSA isolates in CC45 or CC30. By cgMLST analysis, there were 9 allelic differences between two isolates, with the remaining 150 isolates differing from each other by over 40 alleles. The two isolates were retroactively epidemiologically linked by medical record review**.** Overall, cgMLST analysis resulted in higher resolution epidemiological typing than did multilocus sequence or *spa* typing.

## Introduction

*Staphylococcus aureus* is responsible for a high percentage of hospital- and community-acquired infections worldwide. It is also a leading cause of bacteremia, often associated with metastatic infections and significant morbidity and mortality. The epidemiology of *S*. *aureus* infection has changed over the past decade and a half, with methicillin-resistant *S*. *aureus* (MRSA) being increasingly identified in community settings. This has led to interest in attempting to understand the genetic background of the pathogen across different geographic regions and care settings [1–3]. Since a retrospective, population-based, cohort study was done to evaluate initial episodes of *S*. *aureus* bacteremia (SAB) occurring in adult residents of Olmsted County, Minnesota, from 1998 through 2005 [4], there has been little data about the epidemiology of SAB from this area, and there has been no genetic characterization of involved isolates. In hospital settings, *S*. *aureus* may be transmitted from patient to patient via healthcare worker hands, contaminated equipment or through environmental contamination. Focused infection prevention and control (IPAC) measures guided by epidemiological investigations are necessary to prevent nosocomial transmission. To inform IPAC strategies within individual institutions, it is helpful to understand the molecular epidemiology of infection, including whether particular strains are prevalent, and whether or not there is evidence of patient to patient transmission.

In recent years, numerous tools have become available for typing of *S*. *aureus*, ranging from fingerprint-based methods such as pulsed-field gel electrophoresis (PFGE), to PCR-based methods such as multilocus variable-number tandem repeat analysis, to sequence-based methods such as multilocus sequencing typing (MLST), and most recently, to whole genome sequencing (WGS) [5]. The most widely used molecular typing method for defining MRSA epidemiology has traditionally been PFGE [6]. However, PFGE results can be challenging to compare between laboratories and to interpret; furthermore, PFGE is low throughput, is not suitable for long-term epidemiological investigations and assesses a limited amount of the microbial genome [7, 8]. A low mutation rate of the sequence fragments of seven housekeeping genes makes MLST most suitable for long-term and global epidemiological studies [9]. DNA sequencing of short sequence repeats of the polymorphic X region of the protein A gene, *spa*, consisting of a variable number of 21- to 27-bp sequences, is an alternative method for typing *S*. *aureus* [10, 11]. For MRSA, characterization of the resistance-conferring mobile genetic element, SCC*mec* is another method of typing. Recently, WGS-based typing has been made possible through next-generation DNA sequencing (NGS) technology; this approach allows detection of single nucleotide polymorphisms and core genome MLST (cgMLST), among analytical approaches. Through mapping of genome-wide variations, this approach theoretically provides optimal resolution to infer phylogenetic relatedness. WGS has been used in evolutionary studies, outbreak investigations, and phylogeographic distribution analyses [12–14].

The aim of the present study was to use WGS to investigate the molecular epidemiology of *S*. *aureus* isolates causing bacteremia in a single large Minnesota medical center. We also compared *spa* typing using data extracted from WGS to PCR/Sanger sequencing-based *spa* typing, and WGS-generated MLST- to cgMLST-based typing.

## Materials and methods

### Patient population

All episodes of *S*. *aureus* bacteremia in patients ≥16 years of age at the Mayo Clinic, in Rochester, Minnesota (2,059 hospital beds) from January 2015 through December 2015 were considered for inclusion. Isolates were prospectively collected. Only the first episode was analyzed in cases of recurrent SAB during the study period. Isolates associated with polymicrobial infection or suspected contamination, as well as those with insufficient medical record data were excluded. This non-interventional study was approved by the Mayo Clinic Institutional Review Board with a waiver of informed consent (IRB 15–001990).

### Data collection and definitions

Patient demographics and clinical characteristics were recorded. Community-onset infections were defined as those either present or incubating at the time of hospital admission, or for which the first positive culture was obtained within 48 hours of admission. Community-onset infections were further classified as healthcare-associated (HCA) or community-associated (CA). If a patient fulfilled any of the following criteria, SAB was classified as HCA [15, 16]: (1) presence of an invasive device at the time of admission or at onset of infection; (2) history of MRSA infection or colonization in the antecedent 6 months; (3) history of surgery, hospitalization, dialysis, or residence in a long-term care facility in the antecedent 12 months; or (4) use of systemic antibiotics within 3 months prior to SAB. A case was classified as hospital onset (HO) if the first *S*. *aureus* blood culture was obtained 48 hours or more after hospital admission.

### Identification and antimicrobial susceptibility testing

The BD BACTEC^™^ FX system (Becton, Dickinson and Company, Franklin Lakes, NJ) was used for blood culture. Blood culture bottles showing possible staphylococci were tested with the FilmArray^®^ BCID panel (bioMérieux, Marcy l’Étoile, France), which includes rapid detection of *mec*A and *S*. *aureus*. Identification of *S*. *aureus* was by standard methods (biochemicals or matrix-assisted laser desorption ionization time-of-flight mass spectrometry); antimicrobial susceptibility testing was performed using agar dilution, with results interpreted using the Clinical and Laboratory Standards Institute guidelines [17]. Antibiotics tested included clindamycin, mupirocin, oxacillin, rifampin, trimethoprim-sulfamethoxazole, vancomycin, and daptomycin, with ceftaroline additionally tested against MRSA.

### *spa* typing based on PCR/Sanger sequencing

Staphylococcal DNA was extracted using the QIAamp DNA Mini Kit (Qiagen, Valencia, CA). *spa* typing was performed with PCR followed by Sanger sequencing, as previously described [18]. Assignment of *spa* type and Based Upon Repeat Pattern (BURP) analysis for determination of *spa* clonal complex (*spa* CC) were performed using Ridom StaphType (Ridom GmbH, Germany).

### DNA sequencing for WGS and cgMLST

Staphylococcal DNA was prepared for WGS using lysostaphin or achromopeptidase, the Maxwell^®^ 16 Tissue DNA Purification kit (Promega Corporation, Madison, WI) and the Genomic DNA Clean & Concentrator^™^ kit (Zymo Research Corporation, Irvine, CA), performed according to the manufacturers’ instructions. WGS was performed on a MiSeq (Illumina, Inc., San Diego, CA) using the paired-end mode (2x250) in the Mayo Clinic Medical Genome Facility. Reads were trimmed using Trimmomatic. FASTQ files were imported into SeqSphere+ software version 2.3 (Ridom GmbH) for analysis. *de novo* assembly and application of a *S*. *aureus* cgMLST scheme was done using SeqSphere+ with default parameters and with 1,861 queried target genes, as described previously [19]. If genomes did not contain ≥95% of the 1,861 cgMLST target genes with a minimum of ten times global local coverage, WGS was repeated.

### Determination of *spa*, MLST and SCC*mec* types from WGS data

In addition to performing a specific PCR followed by Sanger sequencing, *spa* type was determined using SeqSphere+ for *spa* typing with the WGS data. The WGS data was also used for MLST and SCC*mec* type determination. For MLST, seven target genes (*arc*C, *aro*E, *glp*F, *gmk*, *pta*, *tpi*, *yqi*L) were analyzed [20], also in SeqSphere+. If there was no matched allele in SeqSphere+, the sequence of the particular gene(s) from the *S*. *aureus* MLST scheme was manually extracted from the WGS data and compared to references in NCBI and the *S*. *aureus* MLST database (http://pubmlst.org/saureus) to assign the classical sequence type (ST). Novel sequences were submitted to the *S*. *aureus* MLST database http://pubmlst.org/saureus. The associated clonal complex (CC) was calculated using the eBURST algorithm (http://saureus.mlst.net/eBURST/), with CCs defined using a criterion of six common alleles [21]. SCC*mec* typing was determined by assessing *ccr* and *mec* gene complexes from WGS data. Sequences have been deposited in the NCBI Sequence Read Archive under bioproject number PRJNA384623.

## Results

Of a total of 176 non-duplicate SAB isolates during the study period, 152 were included after excluding 13 which were associated with polymicrobial infection, 6 which were considered possible contaminants and 3 with insufficient medical record data. Two isolates failed to provide sufficient WGS data after multiple attempts. 63% were from male patients; the mean patient age was 61±16.8 (± standard deviation) years. Forty eight (32%) were methicillin–resistant, both phenotypically and by detection of *mec*A. Three were CA-MRSA, 25 HCA-MRSA, 20 HO-MRSA, 31 CA-methicillin-susceptible *S*. *aureus* (MSSA), 54 HCA-MSSA, and 19 HO-MSSA.

### Comparison of antimicrobial susceptibility of MSSA and MRSA

54% of MRSA isolates and 14% of MSSA isolates were resistant to clindamycin. Clindamycin resistance was present 6% of CA isolates, 27% of HCA isolates, and 44% of HO isolates. Fewer than 5% of MRSA isolates were resistant to rifampin, mupirocin, or trimethoprim-sulfamethoxazole (Table 1). All MRSA isolates were susceptible to vancomycin, daptomycin, and ceftaroline.

**Table 1:** Antimicrobial resistance of 152 *Staphylococcus aureus* bacteremia-associated isolates.

Antimicrobial agent	MSSA N = 104 (%)	MRSA N = 48 (%)	Total N = 152 (%)
Oxacillin	0	48 (100)	48 (32)
Clindamycin	14 (14)	26 (54)	40 (26)
Rifampin	0	1 (2)	1 (1)
Mupirocin	1 (1)	1 (2)	2 (1)
Trimethoprim/sulfamethoxazole	1 (1)	2 (4)	3 (2)
Vancomycin^a^	0	0	0
Daptomycin	0	0	0
Ceftaroline	Not tested	0	

MSSA, methicillin-susceptible *S*. *aureus*; MRSA, methicillin-resistant *S*. *aureus*

^a^ Among MSSA, 1, 75 and 28 had vancomycin MICs of <1, 1, and 2 μg/ml, respectively, and among MRSA, 1, 24 and 23 had vancomycin MICs of <1, 1, and 2 μg/ml, respectively.

### *spa* typing, including a comparison between PCR/Sanger sequencing- and WGS-based *spa* typing

**Fig 1:**
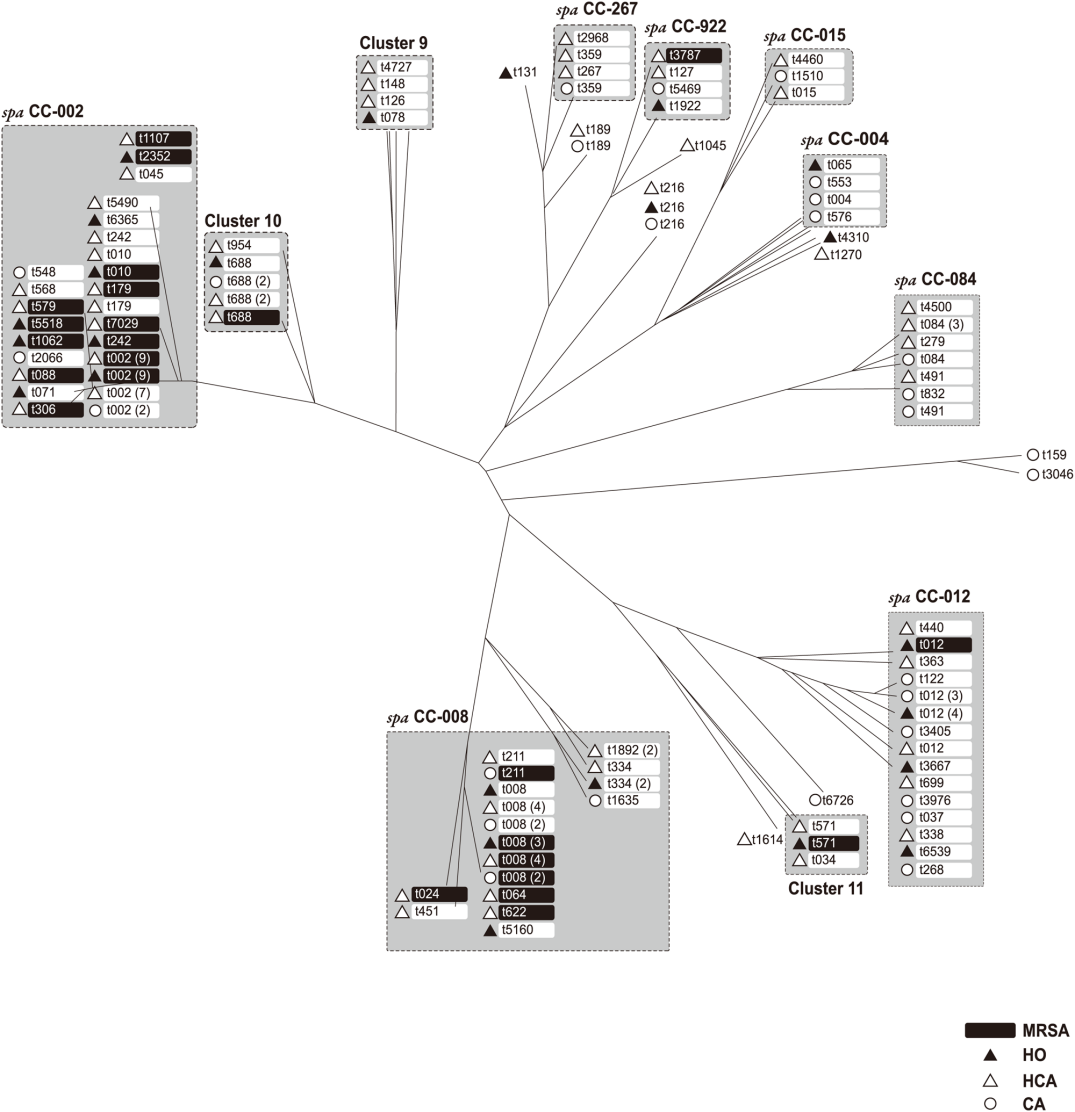
*spa* clustering of 148 *Staphylococcus aureus* bacteremia isolates using PCR/Sanger sequencing-based *spa* typing. MRSA, methicillin-resistant *S*. *aureus*; HO, hospital onset; HCA, healthcare-associated; CA, community-associated. Values in parentheses are the numbers of isolates of a given *spa* type.

Using PCR/Sanger sequencing-based *spa* typing, there were 66 *spa* types, clustered into 11 *spa* clonal complexes (CCs) and 10 singleton *spa* types (Fig 1). Four isolates (t457, t586, t2465, t3736) were non-typeable as a result of their having fewer than five repeats.

*spa* typing of MSSA revealed wide genotypic diversity, with 61% distributed into four *spa* CCs (CC-002, CC-012, CC-008 and CC-084, Table 2). Of the 48 MRSA isolates, 42 (88%) belonged to *spa* CC-002 or CC-008.

**Table 2:** Genotypic characteristics of *Staphylococcus aureus* study isolates.

	MSSA (N = 104)		MRSA (N = 48)	
*spa* CC^a^	No. of *spa* types (ST)	No. of isolates (%)	No. of *spa* types (ST)	No. of isolates (%)
CC-002	11 (t002, t010, t045, t071, t179, t242, t548, t568, t2066, t5490, t6365)	19 (18)	12 (t002, t010, t088, t179, t242, t306, t579, t1062, t1107, t2352, t5518, t7029)	29 (60)
CC-012	12 (t012, t037, t122, t268, t338, t363, t440, t699, t3405, t3667, t3976, t6539)	19 (18)	1 (t012)	1 (2)
CC-008	7 (t008, t211, t334, t451, t1635, t1892, t5160)	16 (15)	5 (t008, t024, t064, t211, t622)	13 (27)
CC-084	5 (t084, t279, t491, t832, t4500)	9 (9)		
CC-922	3 (t127, t922, t5469)	3 (3)	1 (t3787)	1 (2)
CC-004	4 (t004, t065, t553, t576)	4 (4)		
CC-267	3 (t267, t359, t2968)	4 (4)		
CC-015	3 (t015, t1510, t4460)	3 (3)		
CCs with no founder	8 (t034, t078, t126, t148, t571, t688, t954, t4727)	12 (12)	2 (t571, t688)	2 (4)
Singletons	10 (t131, t159, t189, t216, t1045, t1270, t1614, t3046, t4310, t6726)	13 (13)		
Not typeable/excluded	2 (t586, t3736)	2 (2)	2 (t457, t2465)	2 (4)
Total	68	104 (100)	22	48 (100)

MSSA, methicillin-susceptible *S*. *aureus*; MRSA, methicillin-resistant *S*. *aureus*

^a^ *spa* Types with fewer than five repeats were excluded. *spa* types were grouped into the same *spa* CC if cost ≤six [11].

Compared to PCR/Sanger sequencing-based *spa* typing, WGS-based *spa* typing revealed 26 isolates with apparently discrepant results. Using SeqSphere+, *spa* genes were not detected in 2 isolates and 24 isolates showed apparently different results than PCR/Sanger sequencing. When sequences were compared manually however, sequence differences were not confirmed for 18 isolates whereas they were confirmed in 6. For the 18 isolates for which the differences were not confirmed, WGS sequencing results showed several nucleotide differences in 12 isolates, and possible insertion, deletion or substitution errors in 6 isolates.

### MLST from WGS

152 isolates were subjected to MLST analysis; one locus was not found in one isolate, which was not further analyzed. Using the *S*. *aureus* MLST database (http://pubmlst.org/saureus), there were 31 sequence types. The most prevalent sequence type was ST5, represented by 36 isolates (24% of all isolates); the next prevalent clone was ST8, represented by 24 isolates (16% of all isolates). Eighteen STs were divided into 6 CCs by eBURST and 13 STs were singletons. The singletons did not belong to any CC, and differed from all STs in the dataset at two or more MLST loci.

All CA-MRSA grouped into CC8 (2 ST8 and 1 ST3342); among HCA- and HO-MRSA (45 isolates), the most common sequence type was ST5 (15 isolates). There were no MRSA in CC45 or CC30. For MSSA, the common CCs were CC5 (23%), CC30 (19%), CC8 (15%), and CC15 (11%). Common MRSA CCs were CC5 (67%) and CC8 (25%).

### SCC*mec* typing from WGS

Two SCC*mec* types were found among the 48 MRSA isolates, type II (56%) and type IV (44%). SCC*mec* type II isolates were all CC5, and SCC*mec* type IV was the most common type in CC8 (57%).

### cgMLST from WGS

**Fig 2:**
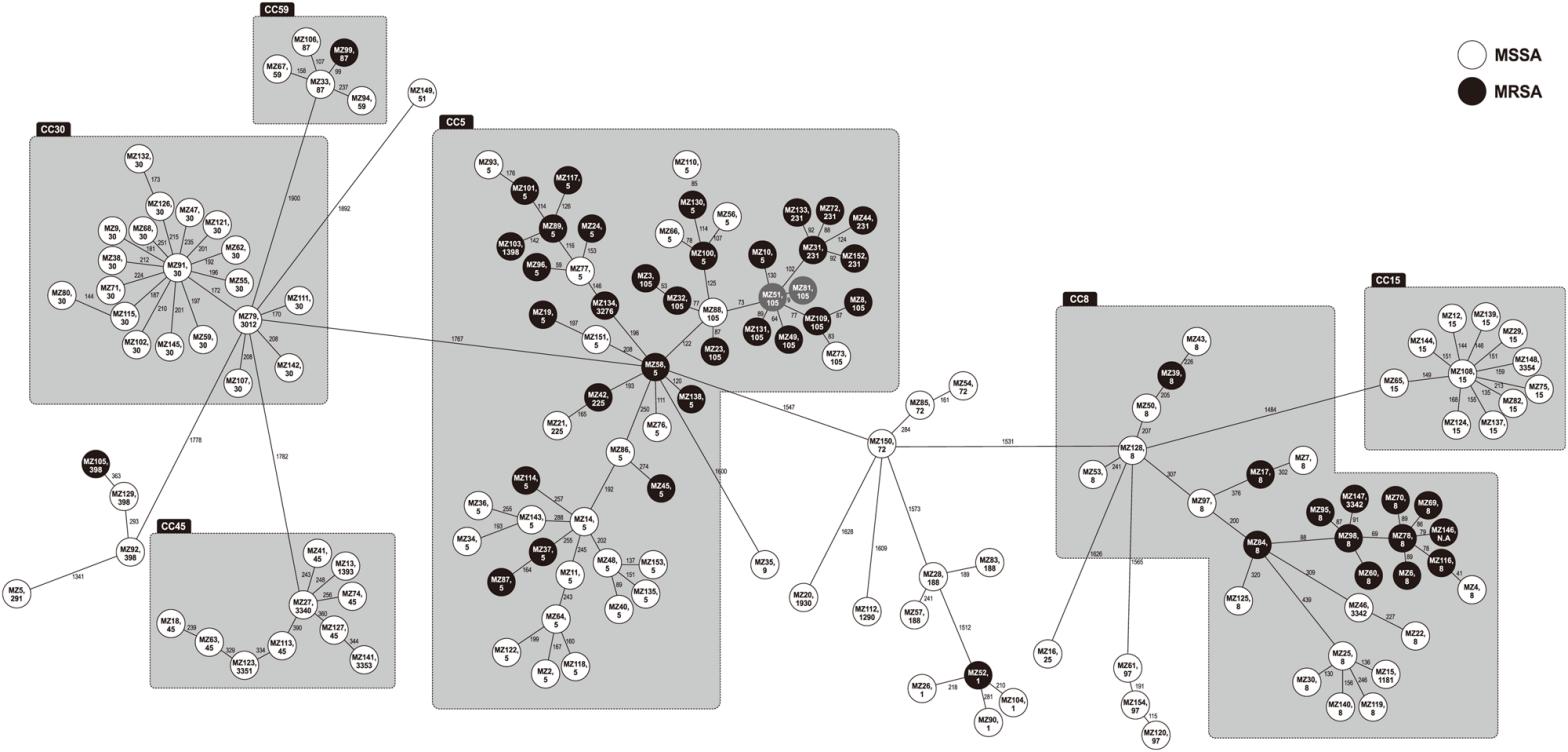
Minimum spanning network built from the core genome MLST allelic profiles of 152 *Staphylococcus aureus* bacteremia isolates with MLST clonal complexes (CCs) overlain. Each circle represents a single genotype, i.e., an allelic profile based on up to 1,861 target genes present in the isolates with the “pairwise ignoring missing values” option turned on in the SeqSphere^+^ software during comparison. The circles are named with the sample ID and MLST ST and color coded as MSSA (white) or MRSA (black). The numbers on the connecting lines indicate the numbers of different alleles between the connected genotypes. MSSA, methicillin-susceptible *S*. *aureus*; MRSA, methicillin-resistant *S*. *aureus*; N.A, not available. The MRSA isolates with 9 allelic differences, MZ51 and MZ81, are highlighted in gray. CC30 by MLST corresponds to *spa* CC-012, CC5 corresponds to *spa* CC-002, and CC8 corresponds to *spa* CC-008.

Fig 2 shows a minimum spanning tree based on the cgMLST allelic profiles with overlaid MLST clonal complex (CC) groupings. cgMLST enabled higher resolution within CCs than did MLST or *spa* typing (Fig 2).

By cgMLST analysis, there were 9 allelic differences between 2 isolates (MZ51 and MZ81), with the remaining 150 isolates differing from each other by over 40 allelic differences. MZ51 and MZ81 were both HO-MRSA bacteremia isolates, recovered from patients with peripheral inserted central catheters. The two bacteremias occurred 55 days apart. The patients from whom the isolates derived were both cared for on the same service and both had care provided by the same nurse practitioner.

**Fig 3:**
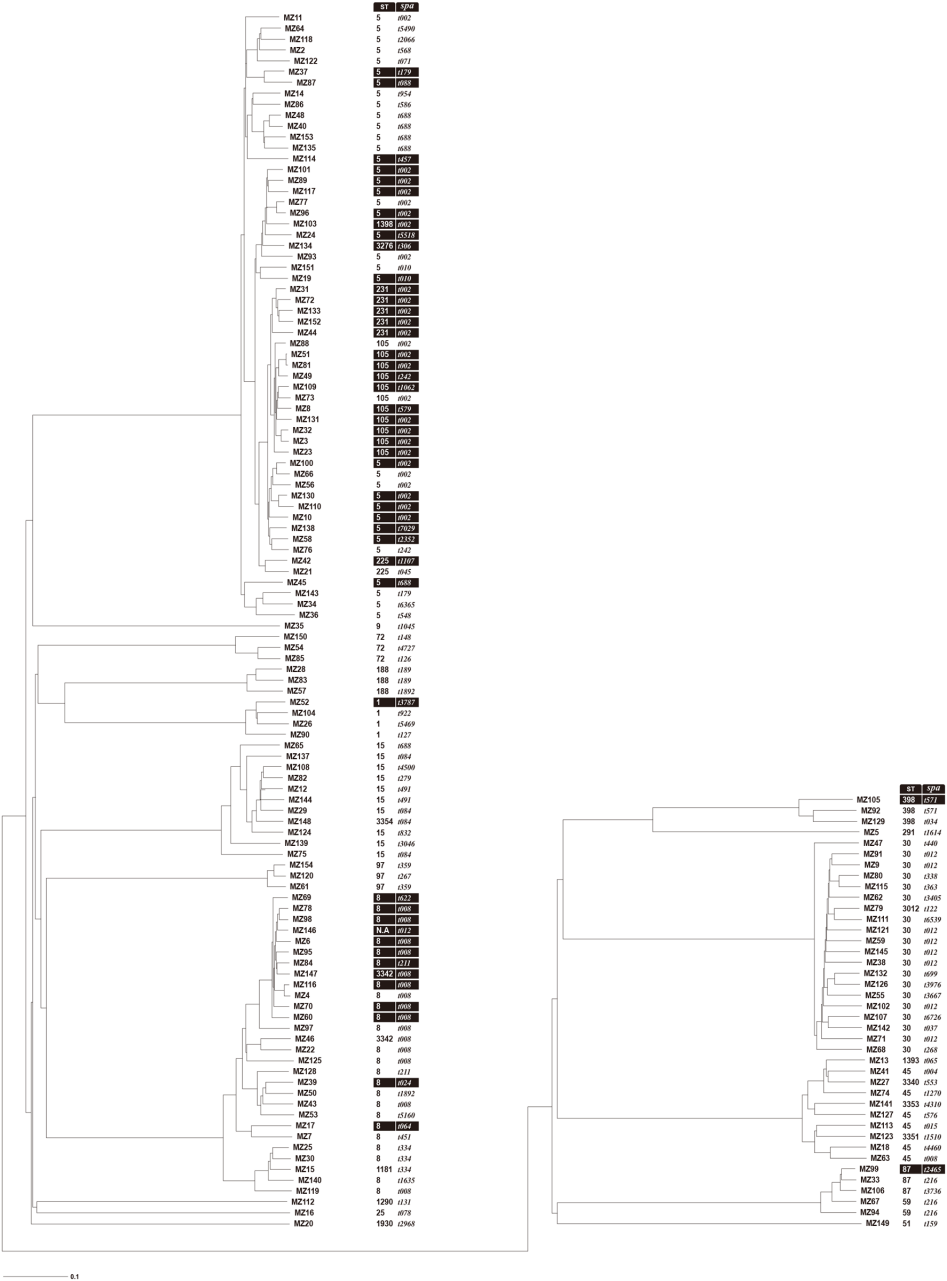
Phylogenetic tree of cgMLST analysis generated using the neighbor-joining method compared to MLST (ST) and traditional *spa* typing (*spa*) for 152 *Staphylococcus aureus* bacteremia isolates. cgMLST analysis was based on allelic profiles as determined using SeqSphere+ software. N.A., not available. Methicillin-resistant isolates are highlighted in black.

A phylogenetic dendrogram of cgMLST data was generated to visualize the relatedness among isolates compared to MLST and PCR/Sanger sequencing-based *spa* typing (Fig 3).

## Discussion

In a single large Minnesota medical center, over 150 cases of SAB occurred in the year 2015, approximately one third of which were MRSA. *spa* typing of MSSA revealed wide genotypic diversity; the common MRSA clones were *spa* CC-002 and CC-008. By MLST analysis, CC45 and CC30 contained only MSSA isolates, and common MRSA clones were CC5 and CC8. cgMLST results yielded higher resolution typing results compared to MLST or *spa* typing, and demonstrated a single episode of possible hospital-based transmission of *S*. *aureus* bacteremia. Although there are no standardized thresholds for interpreting clonality with cgMSLT, we recently proposed that using the scheme applied herein for *S*. *aureus*, ≤8 allelic differences be considered related, 9–29 allelic differences be considered possibly related and 30 or more allelic differences be considered unrelated [22]. The findings presented herein support the use of these thresholds.

Outbreaks of CA-MRSA infections have been described worldwide with successful clones often associated with specific geographical locations [23]. Dominant clones of CA-MRSA in the United States are ST1 (PFGE type USA400) and ST8 (PFGE type USA300), and a common HCA-MRSA clone is ST5 (PFGE type USA100). In our study, only 3 cases were CA-MRSA of which 2 were ST8; the dominant clone for HCA- and HO-MRSA was ST5. Although MRSA isolates were more related with healthcare associated infection, HO MSSA bacteremia was surprisingly frequent. There were three ST398 isolates (t034, t571 in cluster 11 in Fig 1), one of which—MRSA isolate MZ105 (t571)—may have been livestock-associated.

Prevention of *S*. *aureus* bloodstream infections is compromised by incomplete understanding of transmission patterns of *S*. *aureus*, which in turn reflects, in part, suboptimal resolution offered by conventional typing methods. Analysis of every nucleotide in the genome provides a complete inventory of micro-evolutionary changes, but is impractical for large population samples. The use of cgMLST maps single nucleotide polymorphisms and insertion/deletions across a large number of genes to a reference sequence [24, 25]. cgMLST provides higher discrimination than ordinary MLST. In our study, only 2 of 152 isolates had a low number of allelic differences by cgMLST suggesting possible relatedness; these two cases were retroactively determined to be epidemiologically connected. cgMLST was used for the first time with next generation sequencing data for phylogenetic analysis of the German 2011 enterohemorrhagic *Escherichia coli* O104:H4 outbreak [26]. More recently, cgMLST was used to evaluate a meningococcal outbreak [27]. In a neonatal MRSA outbreak [24], a rapid sequencing platform revealed a distinct cluster of outbreak isolates and clear separation between outbreak and non-outbreak isolates with same sequence type (ST22). As the costs of sequencing have begun to approach those of conventional methods, high-resolution typing with WGS is now the state-of-the-art tool for routine epidemiological investigations.

We found that traditional *spa* typing using PCR/Sanger sequencing had better performance than WGS-based *spa* typing. Bletz et al. suggested that extraction of *spa* types from WGS with an optimized *de novo* assembly may enable nearly full compatibility with PCR/Sanger sequencing-based *spa* typing for MRSA [28]. However, assembly of repetitive areas based on short Illumina NGS read data may be prone to misassembling, resulting in failure or errors [29], as illustrated herein. Although it is not possible to compare data directly since different DNA extracts were used for PCR/Sanger sequencing- and WGS-*spa* typing herein, *spa* typing discrepancies between WGS data and traditional *spa* typing caution against WGS usage for *spa* typing. We note that we used a paired-end 250 base pair read length protocol for sequencing and that longer reads may improve WGS-based *spa* typing.

There are several limitations of our study. First, there was some missing data in the cgMLST targets. Second, we analyzed only *S*. *aureus* bacteremia patients and thus we cannot assess nosocomial transmission of non-bacteremia events or non-bacteremia to bacteremia events. Third, for our *spa* typing studies, we did not perform PCR/Sanger sequencing from the same DNA that had been used for WGS.

## Conclusions

cgMLST analysis resulted in higher resolution epidemiological typing than did MLST or *spa* typing. MSSA isolates associated with SAB at a single large Minnesota medical center exhibited wider genotypic diversity than did MRSA isolates. By cgMLST analysis, only two of 152 SAB isolates appeared to be possibly related and conceivably associated with intra-hospital transmission.
